# Ability of pleth variability index to detect hemodynamic changes induced by passive leg raising in spontaneously breathing volunteers

**DOI:** 10.1186/cc6822

**Published:** 2008-03-06

**Authors:** Geoffray Keller, Emmanuel Cassar, Olivier Desebbe, Jean-Jacques Lehot, Maxime Cannesson

**Affiliations:** 1 Hospices Civils de Lyon, Groupement Hospitalier Est, Department of Anesthesiology and Intensive Care, Louis Pradel Hospital and Claude Bernard Lyon 1 University, INSERM ERI 22, 28 avenue du doyen Lépine, 69500 Bron-Lyon, France; 2 Hospices Civils de Lyon, Groupement Hospitalier Est, Department of Cardiology, Louis Pradel Hospital and Claude Bernard Lyon 1 University, 28 avenue du doyen Lépine, 69500 Bron-Lyon, France

## Abstract

**Introduction:**

Pleth Variability Index (PVI) is a new algorithm that allows continuous and automatic estimation of respiratory variations in the pulse oximeter waveform amplitude. Our aim was to test its ability to detect changes in preload induced by passive leg raising (PLR) in spontaneously breathing volunteers.

**Methods:**

We conducted a prospective observational study. Twenty-five spontaneously breathing volunteers were enrolled. PVI, heart rate and noninvasive arterial pressure were recorded. Cardiac output was assessed using transthoracic echocardiography. Volunteers were studied in three successive positions: baseline (semirecumbent position); after PLR of 45° with the trunk lowered in the supine position; and back in the semirecubent position.

**Results:**

We observed significant changes in cardiac output and PVI during changes in body position. In particular, PVI decreased significantly from baseline to PLR (from 21.5 ± 8.0% to 18.3 ± 9.4%; *P *< 0.05) and increased significantly from PLR to the semirecumbent position (from 18.3 ± 9.4% to 25.4 ± 10.6 %; *P *< 0.05). A threshold PVI value above 19% was a weak but significant predictor of response to PLR (sensitivity 82%, specificity 57%, area under the receiver operating characteristic curve 0.734 ± 0.101).

**Conclusion:**

PVI can detect haemodynamic changes induced by PLR in spontaneously breathing volunteers. However, we found that PVI was a weak predictor of fluid responsiveness in this setting.

## Introduction

Hypovolaemia is among the most frequent causes of circulatory failure in the emergency medicine setting. Fluid loading is often the first therapy to be applied to optimize cardiac output (CO) in this situation. Static and the usual clinical variables (central venous pressure, pulmonary capillary wedge pressure, left ventricular end-diastolic area, mean arterial pressure [MAP] and/or tachycardia) are known to be of little value in discriminating between patients who will and those who will not respond to volume expansion [1-5].

On the other hand, dynamic indices that rely on cardiopulmonary interactions (variation in arterial pulse pressure (ΔPP) [3], inferior vena cava diameter [6], superior vena cava diameter [7], stroke volume [8] and aortic blood flow [4]), which are based on variation in left ventricular stroke volume, have been shown to be more accurate predictors of fluid responsiveness in mechanically ventilated patients [2,3,6,8]. However, these indices are invasive, not universally available, or operator dependent.

Respiratory variation in pulse oximeter waveform amplitude (ΔPOP) has been shown to be strongly related to ΔPP [9], to be sensitive to changes in ventricular preload [10] and to be accurate predictors of fluid responsiveness [2]. Recently, a study conducted in spontaneously ventilated volunteers [11] showed that ΔPOP can reflect changes in ventricular preload in spontaneously breathing volunteers. However, ΔPOP cannot easily be calculated and continuously monitored at the bedside

Pleth Variability Index (PVI; Masimo Corp., Irvine, CA, USA) is new software that allows automatic and continuous monitoring of respiratory variations in the pulse oximeter waveform amplitude. This device has already been tested in our institution in mechanically ventilated patients [12]. The aim of the present study was to test the ability of PVI to detect changes in ventricular preload in spontaneously breathing volunteers.

## Materials and methods

This study was conducted in accordance with the ethical standards of our institution and with the Helsinki Declaration of 1975 and revised in 1983. After written informed consent had been obtained, we studied 25 volunteers with no previous arterial hypertension or known cardiac disease, active sepsis and/or cardiac arrhythmias at the time of the study. Each patient was equipped with a pulse oximeter probe (LNOP^® ^Adt; Masimo Corp.) attached at the index of the left hand and wrapped to prevent outside light from interfering with the signal. This pulse oximeter was connected to a Masimo Radical 7 monitor (Masimo SET; Masimo Corp.) with PVI software (version 7.0.3.3). Pulse oximeter plethysmographic waveforms were recorded from this monitor on a personal computer using PhysioLog software (PhysioLog V1.0.1.1; Protolink Inc., Richardson, TX, USA) and were analyzed by an observer who was blinded to other haemodynamic data. An arterial pressure cuff was positioned at the right arm in volunteers in order to measure systolic arterial pressure (SAP), diastolic arterial pressure (DAP) and MAP, as well as heart rate (HR; Solar 6000; General Electric, Milwaukee, WI, USA). Breathing rate was measured clinically by one of the investigators (OD).

### Cardiac output

Cardiac output was assessed using echocardiography (CV 70; Acuson-Siemens Corp., Mountain View, CA, USA). Aortic blood flow was measured using a pulsed wave Doppler beam directed at the level of the aortic valve such that the click of the aortic closure could be observed. The aortic valve area was calculated from the diameter of the aortic orifice (which was considered as constant in all patients [2 cm]) as aortic valve area = π × (aortic diameter/2)^2^. The stroke volume was calculated as stroke volume = aortic valve area × the velocity time integral of aortic blood flow. The CO was calculated as CO = stroke volume × heart rate. The mean of five measurements performed at the end of the expiratory period were used for statistical analysis.

### Pleth Variability Index calculation

PVI is a measure of the dynamic change in perfusion index that occurs during a complete respiratory cycle and has previously been detailed [12]. For the measurement of pulse oximeter oxygen saturation, red and infrared lights are used. A constant amount of light (termed DC) from the pulse oximeter is absorbed by skin, other tissues and nonpulsatile blood, whereas a variable amount of blood (termed AC) is absorbed by the pulsating arterial inflow. For Perfusion Index (PI) calculation, the infrared pulsatile signal is indexed against the nonpulsatile infrared signal and expressed as a percentage (PI = [AC/DC] × 100), reflecting the amplitude of the pulse oximeter waveform. Then, PVI is calculated by measuring changes in PI over a time interval sufficient to include one or more complete respiratory cycles as follows: PVI = ([PI_max _- Pi_min_]/PI_max_) × 100.

### Other haemodynamic measurements

At each step of the protocol the following parameters were recorded: SAP, MAP, DAP, HR, breathing rate, CO, and pulse oximeter oxygen saturation.

### Study protocol

**Figure 1:**
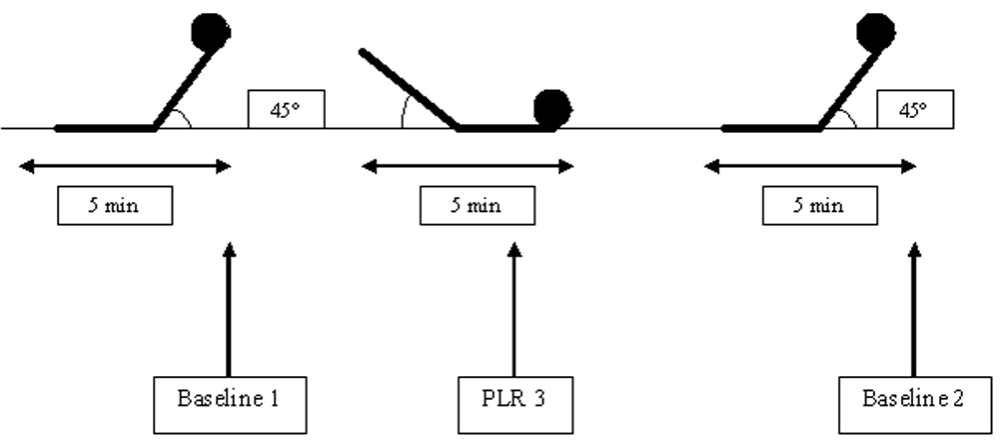
Study protocol. A first set of measurements was taken with volunteers in the semirecumbent position (45°; baseline1 position), when volunteers were quietly and spontaneously breathing after 5 minutes of rest. Then, the lower limbs were lifted straight (45°) with the trunk lowered in the supine position (passive leg raising [PLR] position), and volunteers were left in this position for 5 minutes. A second set of measurements was obtained 3 minutes after leg elevation. We chose not to record data after 1 minute after PLR because we observed significant artefacts in the pulse oximeter waveforms that cast doubt on any interpretation. A third set of measurements was recorded after 5 minutes rest in the semirecumbent position, as in the baseline1 position (baseline2 position). Responders to volume expansion induced by PLR were defined as those volunteers exhibited more than 12.5% [13] increase in cardiac output after PLR.

The study protocol is summarized in Figure 1. A first set of measurements was taken with volunteers in the semirecumbent position (45°; baseline1 position), when volunteers were quietly and spontaneously breathing after 5 minutes of rest. Then, the lower limbs were lifted while straight (45°) with the trunk lowered in the supine position (passive leg raising [PLR] position) and volunteers were left in this position for 5 minutes. A second set of measurements was obtained 3 minutes after leg elevation. We chose not to record data after 1 minute after PLR because we observed significant artefacts in the pulse oximeter waveforms that cast doubt on any interpretation. A third set of measurements was recorded after 5 minutes of rest in the semirecumbent position, as in the baseline1 position (baseline2 position). Responders to volume expansion induced by PLR were defined as volunteers presenting more than 12.5% [13] increase in CO after PLR.

### Statistical analysis

All data are presented as mean ± standard deviation. Changes in haemodynamic parameters induced by changes in loading conditions were assessed using a nonparametric Mann-Whitney U-test or Wilcoxon rank sum test when appropriate. Spearman rank method was used to test linear correlations. Volunteers were divided into two groups according to the percentage increase in CO after PLR: responders were defined as volunteers exhibiting at least a 12.5% [13] increase in CO, and nonresponders were volunteers who exhibited under 12.5% increase in CO. Receiver operating characteristic (ROC) curves was generated for PVI, varying the discriminating threshold of this parameter. *P *< 0.05 was deemed to represent statistical significance. All statistic analysis was performed using SPSS 13.0 for Windows (SPSS, Chicago, IL, USA).

## Results

Twenty-five volunteers were included. This group consisted of 12 females and 13 males aged between 21 and 55 years (mean age 30 ± 9 years).

### Effects of changes in body position on haemodynamic data

**Figure 2:**
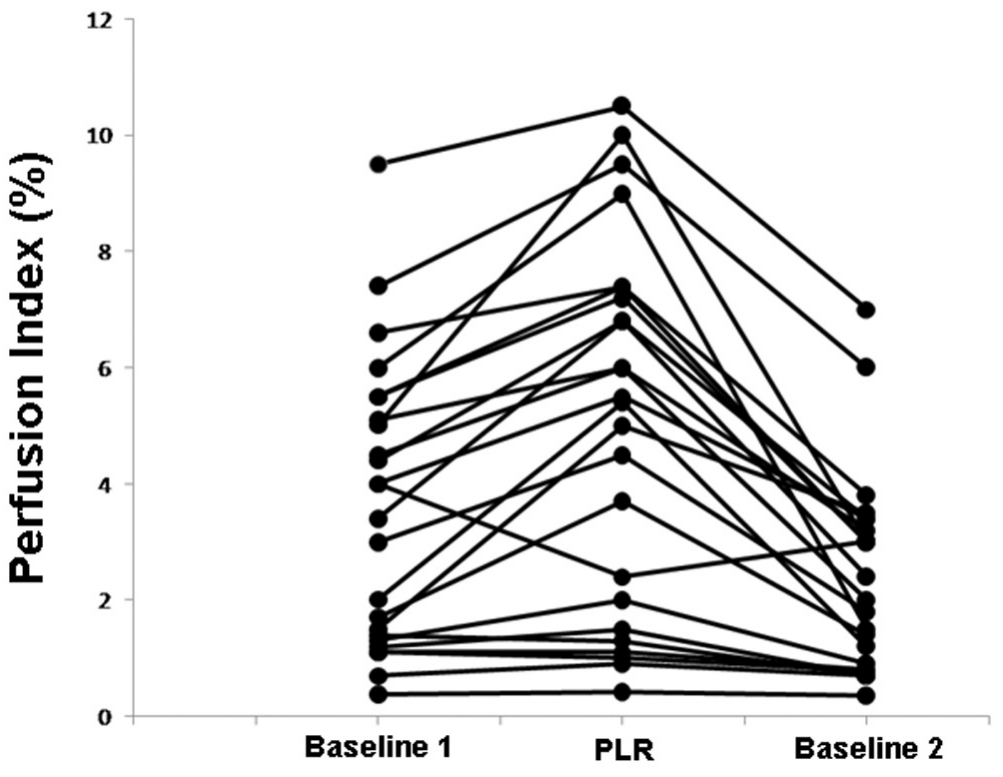
Changes in perfusion index during changes in body position. PLR, passive leg raising.

**Figure 3:**
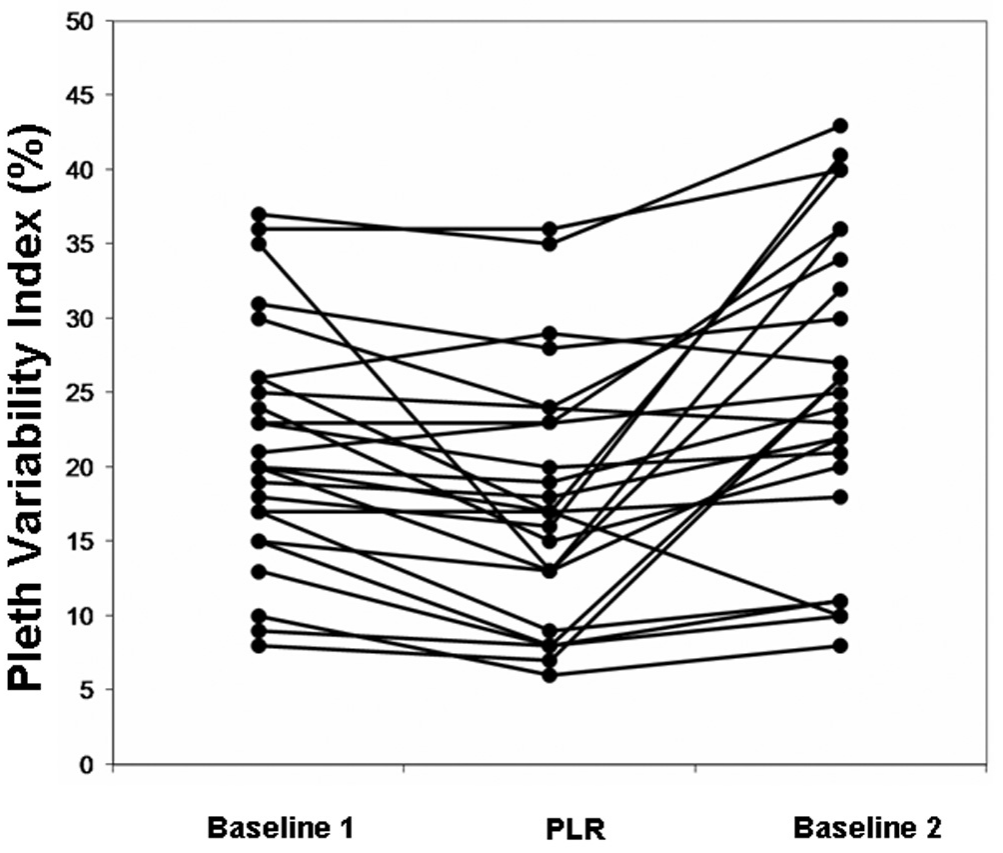
Changes in PVI after changes in body position. PLR, passive leg raising; PVI, Pleth Variability Index.

**Figure 4:**
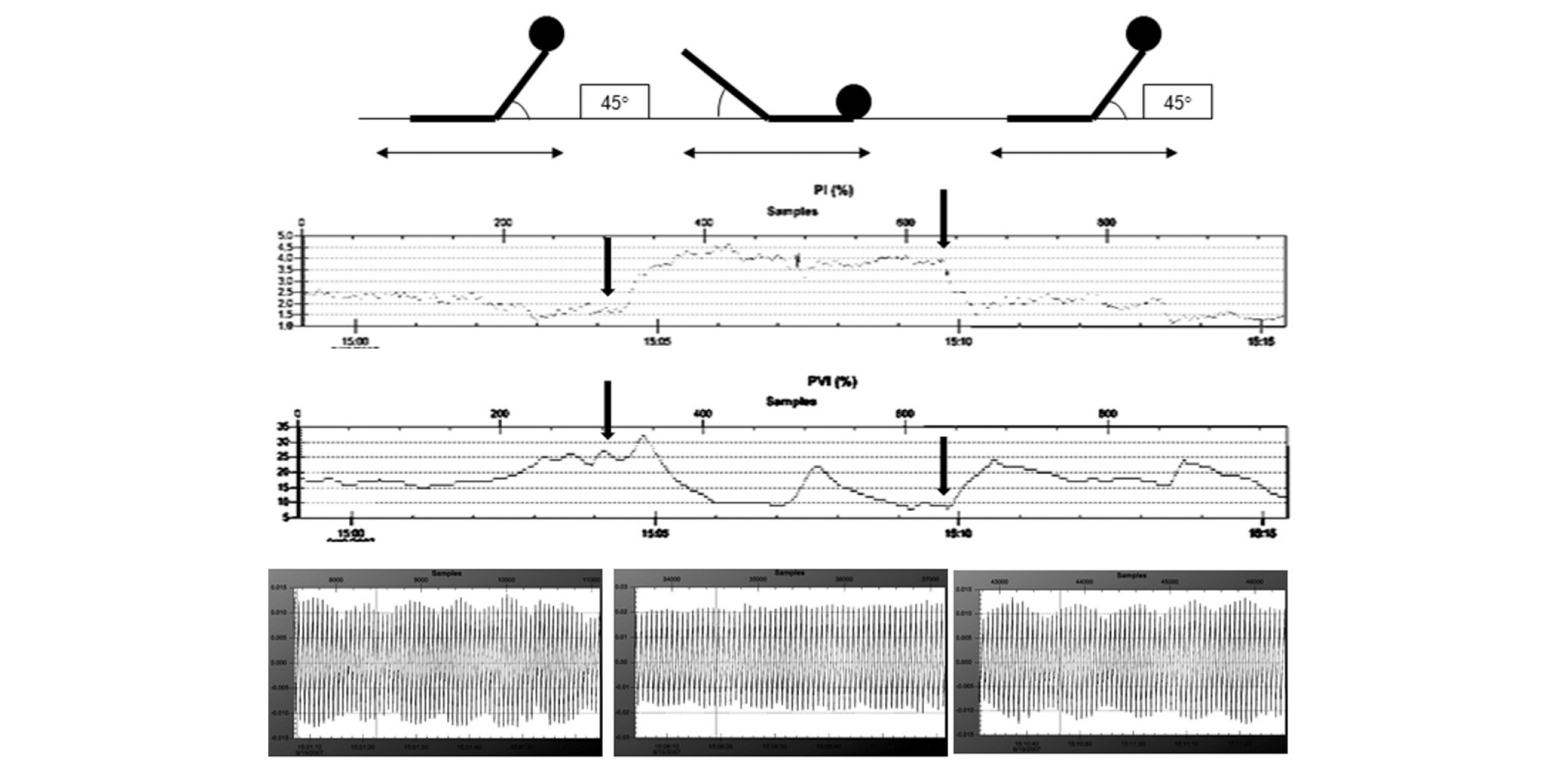
Evolution in PI and PVI. Shown is the volution in Perfusion Index (PI) and Pleth Variability Index (PVI) during changes in body position over a 15-minute period in an illustrative volunteer. Also shown (at the bottom of the figure) are the raw plethysmographic waveforms at baseline1, passive leg raising (PLR), and baseline2. We observed an increase in PI after PLR and a decrease in PI as the volunteer was positioned in the semirecumbent position (baseline 2; see arrows). At the same time, we observed inverse changes in PVI. Specifically, PVI exhibited a slight increase during PLR that was related to a signal artefact in PI. Raw plethysmographic waveforms corroborate PVI values.

Data at baseline, in PLR position and back at the baseline position are shown in Table 1. We observed no significant changes in SAP, DAP, MAP, HR, and breathing rate during changes in body position. In contrast, we observed significant changes in CO, PI and PVI during changes in body position. Specifically, CO was significantly increased from baseline1 to the PLR position (from 4.2 ± 1.1 l/minute to 4.6 ± 1.3 l/minute; *P *< 0.05) and was significantly decreased from PLR position to baseline2 (from 4.6 ± 1.3 l/minute to 3.9 ± 1.1 l/minute; *P *< 0.05). At the same time, we observed a significant increase in PI (from 3.5 ± 2.4% to 4.9 ± 3.2%; *P *< 0.05) and a significant decrease in PVI (from 21.5 ± 8.0% to 18.3 ± 9.4%; *P *< 0.05) from baseline1 to the PLR position, and a significant decrease in PI (from 4.9 ± 3.2% to 2.3 ± 1.7%; *P *< 0.05) and a significant increase in PVI (fom 18.3 ± 9.4% to 25.4 ± 10.6%; *P *< 0.05) from PLR position to baseline2 (Figures 2 to 4).

**Table 1:** Haemodynamic data at baseline, after PLR and back at baseline

Parameter	Baseline1	PLR position	Baseline2
SAP (mmHg)	130 ± 12	125 ± 11	129 ± 11
DAP (mmHg)	73 ± 7	70 ± 6	72 ± 7
MAP (mmHg)	89 ± 8	85 ± 6	89 ± 7
HR (beats/minute)	69 ± 12	69 ± 11	71 ± 11
PP (mmHg)	57 ± 13	59 ± 13	57 ± 8
BR (breaths/minute)	15 ± 5	15 ± 5	15 ± 6
CO (l/min)	4.2 ± 1.1	4.6 ± 1.3*	3.9 ± 1.1^†^
PVI (%)	21.5 ± 8.0	18.3 ± 9.4*	25.4 ± 10.6^†^
PI (%)	3.5 ± 2.4	4.9 ± 3.2*	2.4 ± 1.7^†^

*P < 0.05 versus baseline1; ^†^*P *< 0.05 versus passive leg raising (PLR) position. BR, breathing rate; CO, cardiac output; DAP, diastolic arterial pressure; HR, heart rate; MAP, mean arterial pressure; PI, Perfusion Index; PP, arterial pulse pressure; PVI, Pleth Variability Index; SAP, systolic arterial pressure.

### Ability of PVI to predict fluid responsiveness in spontaneously breathing patients

**Figure 5:**
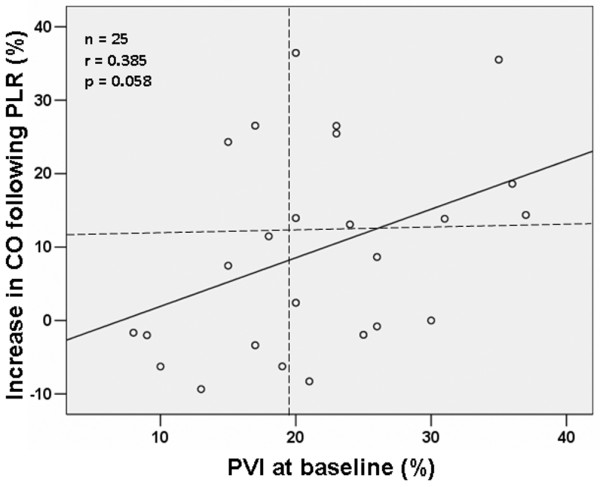
Relationship between PVI at baseline1 and percentage change in CO after PLR. There was non significant relationship between Pleth Variability Index (PVI) at baseline and percentage change in cardiac output (CO) after passive leg raising (PLR). Horizontal dashed line shows increase in CO of 12.5%. Vertical dashed line shows PVI value of 19%, which allowed discrimination between responders and nonresponders to PLR with a sensitivity of 82% and a specificity of 57%.

Of the 25 studied volunteers, 11 (44 %) were responders to PLR. Responders exhibited significantly higher PVI values at baseline1 compared with nonresponders (25.5 ± 7.9 versus 18.3 ± 6.9; *P *< 0.05). A threshold PVI value of >19% was a weak but significant predictor of response to PLR (sensitivity 82%, specificity 57%, area under the ROC curve 0.734 ± 0.101). The relationship between PVI value at baseline and percentage increase in CO after PLR was close to but did not reach statistical significance (*r *= 0.385; *P *= 0.058; Figure 5).

## Discussion

This study shows that PVI, an index that allows automatic and continuous calculation of respiratory variations in the pulse oximeter plethysmographic waveform amplitude, can detect haemodynamic changes induced by passive leg raising in spontaneously breathing volunteers. However, we found that PVI was a weak predictor of fluid responsiveness in this setting, as are most of the dynamic indicators of fluid responsiveness in spontaneously breathing patients.

Assessment of fluid responsiveness in mechanically ventilated patients has now been extensively studied, and it is known that dynamic indicators that rely on cardiopulmonary interactions are the best predictors in this setting [2,3,14]. Recently, ΔPOP has been shown to be a noninvasive and reliable predictor of fluid responsiveness in the operating room [2,15,16] and in the intensive care unit [17] in mechanically ventilated patients. Moreover, it has been demonstrated to decrease significantly after PLR in spontaneously breathing volunteers, suggesting that this parameter may be of value in assessment of fluid responsiveness in this population [11]. However, this index is difficult to measure at the bedside and cannot be visually estimated from the monitor screen because of the gain processing that is used by most of the monitors [2]. We recently demonstrated that PVI could continuously and automatically monitor ΔPOP in mechanically ventilated patients [12]. In that study we found that a PVI value above 11.5% could discriminate between ΔPOP above 13% and ΔPOP of 13% or less with a sensitivity of 93% and a specificity of 97%. Area under the curve for PVI to predict ΔPOP above 13% was 0.990 ± 0.07 in this study. However, although we found PVI to be of value in mechanically ventilated patients, its utility in spontaneously breathing patients had never been investigated [12].

The pulse oximeter waveform relies on light absorption. Briefly, light absorption includes two components. The first component is said to be constant and is due to light absorption by bone, tissue, pigments, nonpulsatile blood and skin. Venous blood is also responsible for some constant absorption, but this is still under investigation [18,19]. The second component is said to be pulsatile absorption, which is due primarily to arterialized blood. The PI is defined as the ratio between constant absorption (AC) and pulsatile absorption (DC), reflecting the amplitude of the plethysmographic waveform. PVI can automatically detect the maximal and minimal PI value over a period of time sufficient to include at least one complete respiratory cycle. PVI is then automatically and continuously calculated as (PI_max _- PI_min_)/PI_max_, reflecting respiratory variations in PI. This algorithm allows continuous monitoring of the respiratory variations in the pulse oximeter waveform amplitude.

Assessment of fluid responsiveness in spontaneously breathing patients is difficult, and cardiopulmonary interactions in this setting differ greatly from those observed in mechanically ventilated patients [20-22]. Moreover, in this setting, frequency and tidal volumes may vary from breath to breath. However, further studies are required to explore this topic, as suggested by recent published experimentations conducted in this setting and focusing on ΔPOP [11]. PLR mimics a 'rapid and transient' fluid loading of 300 ml by transferring a volume of blood to the central compartment. In association with rapid measurements of changes in aortic blood flow, it provides a useful tool with which to evaluate fluid responsivness in mechanically ventilated but also in spontaneously breathing patients who are suspected of being hypovolaemic [13]. In normotensive individuals, this manoeuvre not only increases preload but also decreases peripheral vascular resistance [13]. Our data, showing that PI significantly increases after PLR (Figure 4), may support this hypothesis because PI is related to vasomotor tone. In the present study, we applied a modified form of PLR associated with trunk lowering, which has previously been used and should amplify the transient haemodynamic changes [13]. These changes occur maximally during the first minute and disappear after a few minutes. We performed measurements only during the third minute in order to obtain a stable and reliable plethysmographic signal that was not disturbed by changes in vasomotor tone.

Recently, Soubrier and coworkers [21] found ΔPP to be a weak predictor of fluid responsiveness in spontaneously breathing patients. In that study they showed that ΔPP above 12% was able to discriminate responders from nonresponders to volume expansion with 92% sensitivity and 63% specificity. These data indicate slightly better performance than suggested by our data obtained with PVI. In particular, area under the ROC curve for ΔPP was 0.81 ± 0.08 in their study as compared with 0.734 ± 0.101 in ours. This difference may be related to the sensitivity of the pulse oximeter waveform to changes in vasomotor tone observed in spontaneously breathing volunteers and to the fact that PVI is unable to discriminate between respiratory changes in PI from other changes in PI. We can postulate that these changes are less frequent and less important in mechanically ventilated patients, as was suggested by a previous study conducted in this setting in our institution and showing that PVI was an accurate monitoring of ΔPOP [12]. Further studies investigating the ability of PVI to predict fluid responsiveness in mechanically ventilated and spontaneously breathing patients are warranted.

### Study limitations

We did not conduct real volume expansion in this study. Rather, we chose to use the previously published threshold value of 12.5% increase in CO after PLR as a predictor of fluid responsiveness in spontaneously breathing volunteers [13]. Future studies are planned to assess the ability of PVI to predict fluid responsiveness in the operating room and in spontaneously breathing patients. Moreover, the aim of our study was to describe the changes in PVI after PLR, as was previously done with ΔPOP [11].

As with any other dynamic indicators of fluid responsiveness, PVI cannot be used in patients with cardiac arrhythmias.

We did not assess systemic vascular resistance in our sample of volunteers. However, POP waveform amplitude relies upon this parameter. Consequently, our results cannot be exptrapolated to situations in which systemic vascular resistances are different, such as patients receiving vasoactive drugs.

## Conclusion

PVI, a new parameter that allows automatic and continuous monitoring of the respiratory variations in the pulse oximeter plethysmographic waveform amplitude, can detect haemodynamic changes induced by PLR in spontaneously breathing volunteers. However, its ability to predict fluid responsiveness in spontaneously breathing patients is weak, and consequently whether it should be used to guide volume expansion in this setting is uncertain.

## Key messages

• PVI is a novel parameter that allows automatic and continuous calculation of the respiratory variations in the pulse oximeter waveform amplitude.

• PVI can automatically and noninvasively detect changes in ventricular preload induced by PLR in spontaneously breathing volunteers.

• Changes in preload induced by PLR also induced changes in PI. However, PI was unable to predict increase in CO induced by PLR.

• PVI, as does any other dynamic indicator, appears to be a significant but weak predictor of fluid responsiveness in spontaneously breathing individuals.

• Acute changes in vasomotor tone may influence PVI; hence, this parameter should be interpreted with caution in this setting.

## Abbreviations

AC = alternating current; CO = cardiac output; CVP = mean arterial pressure; DAP = diastolic arterial pressure; DC = direct current; ΔPOP = variation in pulse oximeter waveform amplitude; ΔPP = variation in arterial pulse pressure; HR = heart rate; PI = Perfusion Index; PLR = passive leg raising; PVI = Pleth Variability Index; ROC = receiver operating characteristic; SAP = systolic arterial pressure.

## Competing interests

Software and hardware were provided by Masimo Corp.

## Authors' contributions

GK was responsible for analysis and interpretation of data, and drafting of the manuscript. EC interpreted data and drafted the manuscript. OD interpreted of data and drafted the manuscript. J-JL revising the manuscript critically for important intellectual content and edited the manuscript. MC conceived and designed the study, analyzed and interpreted the data, and edited the manuscript. All authors read and approved the final manuscript.
